# Anomalous Hypothalamic Responses to Humor in Cataplexy

**DOI:** 10.1371/journal.pone.0002225

**Published:** 2008-05-21

**Authors:** Allan L. Reiss, Fumiko Hoeft, Adam S. Tenforde, Wynne Chen, Dean Mobbs, Emmanuel J. Mignot

**Affiliations:** 1 Center for Interdisciplinary Brain Sciences Research (CIBSR), Department of Psychiatry and Behavioral Sciences, Stanford University School of Medicine, Stanford, California, United States of America; 2 Howard Hughes Medical Institute, Center for Narcolepsy, Department of Psychiatry and Behavioral Sciences, Stanford University School of Medicine, Stanford, California, United States of America; 3 MRC-Cognition and Brain Sciences Unit, Cambridge, United Kingdom; University of Southern California, United States of America

## Abstract

**Background:**

Cataplexy is observed in a subset of patients with narcolepsy and affects approximately 1 in 2,000 persons. Cataplexy is most often triggered by strong emotions such as laughter, which can result in transient, yet debilitating, muscle atonia. The objective of this study was to examine the neural systems underlying humor processing in individuals with cataplexy.

**Methodology/Principal Findings:**

While undergoing functional Magnetic Resonance Imaging (fMRI), we showed ten narcolepsy-cataplexy patients and ten healthy controls humorous cartoons. In addition, we examined the brain activity of one subject while in a full-blown cataplectic attack. Behavioral results showed that participants with cataplexy rated significantly fewer humorous cartoons as funny compared to controls. Concurrent fMRI showed that patients, when compared to controls and in the absence of overt cataplexy symptoms, showed pronounced activity in the emotional network including the ventral striatum and hypothalamus while viewing humorous versus non-humorous cartoons. Increased activity was also observed in the right inferior frontal gyri -a core component of the inhibitory circuitry. In comparison, the one subject who experienced a cataplectic attack showed dramatic reductions in hypothalamic activity.

**Conclusions:**

These findings suggest an overdrive of the emotional circuitry and possible compensatory suppression by cortical inhibitory regions in cataplexy. Moreover, during cataplectic attacks, the hypothalamus is characterized by a marked decrease in activity similar to that observed during sleep. One possible explanation for these findings is an initial overdrive and compensatory shutdown of the hypothalamus resulting in full cataplectic symptoms.

## Introduction

Cataplexy, a pathognomonic symptom of narcolepsy, has fascinated clinicians and researchers alike since its initial description in the late 1870s [1]. Symptoms of cataplexy can vary considerably, ranging from a mild sensation of flotation, day time sleepiness and abnormal rapid eye movement to complete muscle paralysis lasting up to several minutes [2]. Because it is associated with a loss of monosynaptic reflexes and is suppressed by drugs known to inhibit Rapid Eye Movement Sleep (REM) sleep (e.g. antidepressant), the symptom is believed to be an equivalent of REM sleep atonia. The symptoms of cataplexy can occasionally occur suddenly and without any obvious trigger, however, joking and humor-related laughter are considered the most common cause of cataplectic attacks [3]. Although there seems to be a strong relationship between the brain regions associated with humor and cataplexy, it is not fully understood why humor causes debilitating muscle atonia in patients with narcolepsy-cataplexy.

To date, several studies have shown that the neural systems associated with reward and emotion are engaged when subjects find cartoons humorous [4]–[7]. These regions extend from the nucleus accumbens to other territories of the emotion network including the amygdala and hypothalamus. Together, these regions are thought to elicit the euphoric feeling associated with finding a joke humorous [4], [6]–[8]. Interestingly, the key neurobiological marker of narcolepsy-cataplexy is the loss of hypocretin (otherwise known as orexin) which is highest (92.5%) in patients who are HLA-DQB1*0602 positive and report cataplectic attacks triggered by laughter [9]. Hypocretin is expressed in a subset of neurons in the hypothalamus, a region associated with both emotion and sleep regulation [10], [11].

We sought to examine brain activation patterns in narcolepsy-cataplexy patients when shown humorous material [12]. We hypothesized that relative to healthy controls, narcolepsy-cataplexy individuals would show abnormal activity in brain regions associated with affect regulation and the hypocretin system - namely the hypothalamus. To test this prediction, we showed healthy subjects and cataplexy patients (Table 1) humorous cartoons while they underwent functional magnetic resonance imaging (fMRI). The analysis of a patient who experienced a prolonged cataplectic attack during scanning gave us the rare opportunity to examine both the non-cataplectic and cataplectic state.

**Table 1:** Demographics and behavioral data.

	Cataplexy	Control
Sample size	10 (7F, 3M)	12 (7F, 5M)
Age (years)	29.8 (6.5)	25.9 (4.1)
% Cartoons Rated Humorous *	23.6 (10.3)	35.5 (6.3)
% Humorous Cartoons Rated Humorous *	48.7 (21.6)	73.6 (11.9)
Intensity Rating of Humorous Trials rated Humorous 1 **	4.4 (1.7)	6.7 (0.7)

1 Ratings on 1–10 scale

* p<0.01

** p<0.001

## Results

### Behavioral Results

#### Group analysis

Patients with narcolepsy-cataplexy rated significantly fewer humorous cartoons as funny compared to controls (t_(20)_ = 2.96, p = 0.008, Table 1). Further, the average post-scan intensity rating for cartoons rated as humorous (i.e., ratings between 1 and 10), was also significantly lower in the cataplexy group compared to controls (t_(20)_ = 3.53, p = 0.002).

#### Cataplectic Attack Patient

In one individual, a humorous event rated 10, triggered a cataplectic attack (“Attack” trial). Further evidence of cataplexy was the inability to make a motor response in subsequent trials.

### Brain Imaging Results

#### Group analysis

**Figure 1:**
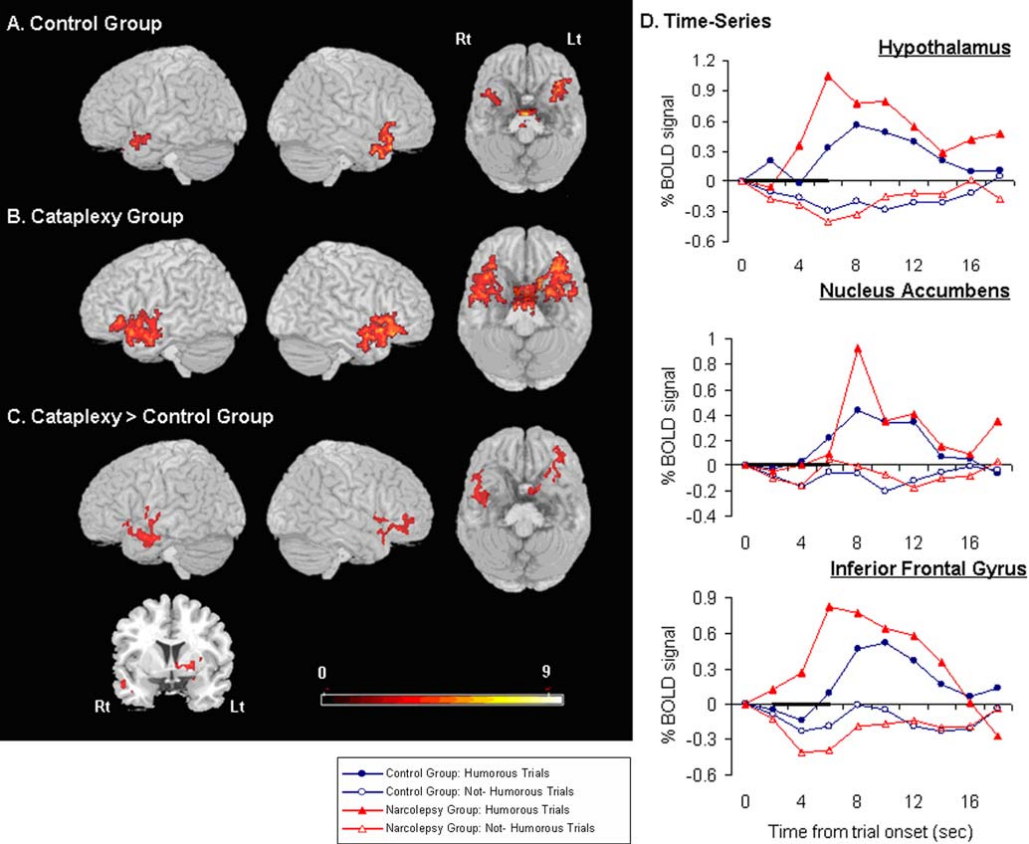
Three-dimensional brain renderings of humorous minus non-humorous trials. (a) Control group. (b) Cataplexy group. (c) Cataplexy group greater than Control group. No regions showed significantly greater activation for the Control compared to the Cataplexy group. p = 0.01 corrected. Scale bar represents t-values. Activation 30 mm below the cortical surface is projected to the surface. (d) Mean time-courses in the hypothalamus, nucleus accumbens and right inferior frontal gyrus.

Analysis of the fMRI data showed control subjects and narcolepsy-cataplexy patients to have significant activation (i.e., changes in oxygenated and deoxygenated blood-flow otherwise known as the Blood Oxygenated Level Dependent [BOLD] signal) for the comparison in which humorous trials were compared to non-humorous trials (Humorous>Non-Humorous; p<0.01 corrected for multiple comparisons) in several brain regions (Fig. 1a, Table 2a). These included cortical structures such as the right inferior frontal gyrus, as well as subcortical and midbrain regions including the thalamus, amygdala, hypothalamus, nucleus accumbens, and ventral tegmental area. (Fig. 1b, Table 2b). These findings support previous studies from our group and others [4], [6]–[8].

**Table 2:** Brain activation profiles for within and between group comparisons for the comparison Humorous>Non-Humorous.

Region		Brodmann Area	t	p	Talairach Coordinates			Cluster Size
					x	y	z	
A. Control Group								
	Left Temporal Pole, Superior Temporal Gyrus, Inferior Frontal Gyrus	38, 47	9.49	<.001	−40	24	−21	363
	Bilateral Hypothalamus, Nucleus Accumbens, Ventral Tegmental Area, Mammillary Body, Left Thalamus, Lentiform Nucleus		8.52	<.001	−2	−12	−11	154
	Bilateral Red Nucleus, Substantia Niagra		4.92	<.001	−2	−18	−6	102
	Rightt Temporal Pole, Superior Temporal Gyrus, Inferior Frontal Gyrus, Amygdala, Uncus	38, 13	4.87	<.001	44	13	−19	125
B. Narcolepsy Group								
	Bilateral Thalamus, Hypothalamus, Nucleus Accumbens, Ventral Tegmental Area, Left Superior Temporal, Inferior Frontal Gyri, Lentiform Nucleus, Uncus	47, 38	8.00	<.001	6	−10	2	2282
	Rightt Fusiform, Superior/Middle Temporal, Inferior Frontal Gyri, Temporal Pole, Insula, Amygdala	13, 20, 21, 38, 47	5.93	<.001	51	−5	−22	1111
	Left Fusiform, Superior/Middle/Inferior Temporal Gyri, Temporal Pole	20, 21, 38	4.63	<.001	−48	−1	−23	116
C. Narcolepsy Group>Control Group								
	Left Lentiform Nucleus, Insula, Claustrum, Nucleus Accumbens, Ventral Tegmental Area, Amygdala, hypothalamus	13, 45	5.01	<.001	−16	6	−4	456
	Right Superior/Middle Temporal, Inferior Frontal Gyrus, Temporal Pole, Insula, Claustrum	13, 20, 21, 38, 47	4.42	<.001	40	−9	−18	452

A direct subtraction (i.e., narcolepsy-cataplexy minus healthy control subjects) demonstrated significantly greater activation (above p<0.01 corrected for multiple comparisons) for narcolepsy-cataplexy patients versus controls for the comparison between Humorous>Non-Humorous comparisons. This greater activation was observed in the emotion network including the nucleus accumbens, hypothalamus and the right inferior frontal gyri (Fig. 1c, Table 2c). There were no regions that showed significantly greater activation for controls compared to the narcolepsy-cataplexy group.

#### Cataplectic Attack Patient

**Figure 2:**
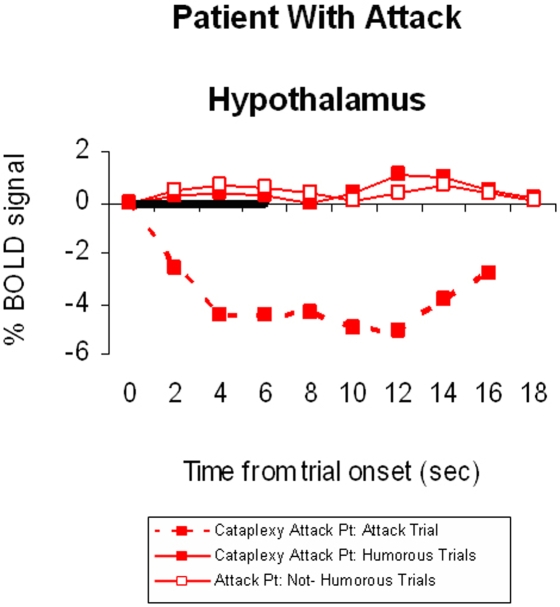
Brain activation profile of cataplexy symptoms in Attack Patient. Time-course in the hypothalamus. Note the overall decreased activity in this region and drastic decrease during cataplectic attack.

Analysis of the cataplectic attack patient showed overall low activity during humorous trials and particularly decreased activity in the hypothalamus during the attack, which coincided with a humorous trial (Figure 2). This was significantly different from the other humorous trials in the attack patient.

## Discussion

In summary, the narcolepsy-cataplexy patients rated the cartoons as being less humorous than the healthy control group. Results from the brain imaging analysis showed that although both groups activated regions associated with humor appreciation, the cataplexy group showed significantly more activity in the nucleus accumbens and hypothalamus as well as the right inferior frontal gyri when directly compared to the healthy control group. Intriguingly, examination of the one patient experiencing the cataplectic attack showed dramatic reductions in hypothalamic activity. Despite the paradoxical nature of this finding (between the cataplexy group and the single cataplectic attack patient), one could suggest that hyperactivation of certain parts of the emotion circuitry lead to subsequent shutdown of the hypothalamus resulting in the characteristic REM-sleep like state.

It is know that the limbic network, including the ventral striatum, hypothalamus, and amygdala, show increased activation during laughter and rapid-eye-movement (REM) sleep (e.g. [13]). It is also notable that the hypothalamus, where hypocretin neurons are located, is also likely involved in the generation of laughter as gelastic seizures are commonly observed with hypothalamic hamartomas [14]. Thus, it is of particular interest that patients with cataplexy showed greater activation in these regions relative to healthy controls with humor stimulation. We have previously observed that the degree of humor rated by healthy subjects is positively correlated with activation of these neural systems [4]. In this respect, the observation of greater activation in subjects with narcolepsy-cataplexy is paradoxical in light of the relatively lower humor ratings by this group. One explanation for this is that the lack of hypocretin in patients with cataplexy intensifies humor-induced activation of these regions, possibly exacerbating normal physiological reactions to humor.

Intriguingly, results from single photon emission computed tomography (SPECT) studies of cataplexy have shown abnormal perfusion in the hypothalamus and basal ganglia. For example, Chabas et al. [15], found restricted hyperperfusion of the cingulate, orbitofrontal and right temporal cortices, and putamen in comparison to a baseline, non-cataplectic state in affected individuals. Hong et al. [16] used a similar technique in two cataplexy patients and found hyperperfusion of the amygdala and basal ganglia (as in [15]), but also hypoperfusion of prefrontal cortex during cataplectic attack. While these results are restricted by low sample size and poor spatial and temporal resolution of SPECT, they suggest that cataplexy is produced by hyperactivation and hypoactivation of amygdalo-cortico-basal ganglia–brainstem circuit.

Our results are in partial support of a recent fMRI study of humor in narcolepsy-cataplexy patients [17]. In that study, and similar to the results presented here, participants with narcolepsy-cataplexy showed heightened activity of the amygdala and nucleus accumbens when exposed to humorous stimuli. Unlike controls however, these subjects did not show altered activity in the hypothalamus in response to humorous (or non-humorous) trials. Abnormal function of the hypothalamic-amygdalar circuit was proposed as one route from which positive emotions may trigger catapletic attacks [17]. Findings in our cataplectic attack patient support this contention, and further suggest that massive suppression of hypothalamic activity may an essential component of a cascade of neural events leading to muscle atonia. Collectively, these fMRI studies of cataplexy indicate that full blown attacks result from an inability to control the emotion system, including the hypothalamus.

The fact that the narcolepsy-cataplexy group showed relatively greater activation of right inferior frontal gyrus, a region known to be involved in inhibitory control [18] is important in the context of the behavioral results. The right inferior frontal gyrus receives direct input from the striatum [19] and is part of the striatal-thalamocortical loop. Anecdotal observations support the notion that cataplexy patients train themselves to suppress laughter and avoid humorous material. This suggests that the right inferior frontal gyrus is involved in the conscious effort to prevent the expression of humor associated behaviors [20], [21] and keeping emotional material out of mind [22]. Decreased activation of the prefrontal cortex during status cataplecticus has also been reported [16]. The right inferior frontal gyrus also modulates the emotional network including the nucleus accumbens and hypothalamus [23], [24]. In this context, it is tempting to speculate that the activation of one or more components of the inhibition “network” serves to prevent exacerbation of normal physiological reactions to humor such as transient muscle weakness reported by healthy subjects when laughing [12], and that patients with cataplexy further activate this compensatory mechanism to prevent cataplexy.

In conclusion, we demonstrate that patients with narcolepsy-cataplexy show overactivation of the nucleus accumbens and hypothalamus. In addition, these subjects also showed increased activation of the inhibitory network which might account for lower ratings of humorous stimuli and suppression of laughter. These findings therefore suggest that increased activation of the nucleus accumbens and hypothalamus might contribute to the symptoms (e.g. muscle atonia) observed in this population, while the inhibition network acts to compensate and may represent top-down cortical control of cataplectic symptoms. Whether severe cataplexy symptoms are marked by a “overactivation” and “shut-down” of the hypothalamus remains an important question for future research.

## Materials and Methods

### Subjects

All subjects with narcolepsy reported clear symptoms of cataplexy, triggered by typical emotions such as laughing and joking, a positive Multiple Sleep Latency Test and met diagnostic criteria for narcolepsy (with cataplexy), as defined by the revised International Classification for Sleep Disorders [25]. All subjects were HLA-DQB1*0602 positive and six of these subjects showed hypocretin values below 110 pg/ml, indicating hypocretin deficiency diagnostic of narcolepsy [26]. Initially, 12 cataplexy patients were included in the study. One cataplexy patient was excluded due to excessive motion during the scan and another due to a self-reported cataplectic attack leaving ten subjects (age: 29.8±6.5, 7 females, 1 left-handed; Table 1).

None of the participants had experienced neurological or psychiatric disorders (other than narcolepsy with cataplexy) and were not currently taking any medications. Six subjects had been treated with anti-narcoleptic treatments (stimulants, antidepressants, sodium oxybate), and in these cases they were withdrawn from these mediations for at least 5 times the half-life of the longest half-life of the medication or five days, whichever was longer. Subjects had no contraindications to MRI. In addition, 12 age-matched healthy adult subjects were scanned (mean age ± standard deviation (SD): 25.9±4.1, 7 females, 0 left-handed). The 11^th^ patient (“Attack” patient) was an 18 year-old, right-handed, healthy male.

There were no significant group differences for age or gender (age t_(20)_ = 1.46, p = 0.16; gender chi-square_(1)_ = 0.78, p = 0.38). The study was approved by the Stanford University Panel on Human Subjects in Medical Research. Written informed consent was obtained according to the Declaration of Helsinki for participation from each subject.

### Experimental Stimuli and Design

The experimental paradigm is identical to that used in previous studies [4], [5], [8]. Subjects were presented 70 stimuli (30 humorous and 40 not humorous cartoons), based on subjective ratings of individuals of similar background, age, and without any known psychiatric disorders. Each cartoon was presented for 6000 ms in an event-related fMRI paradigm in a randomized order. The task was presented using PsyScope 1.2.5 [27]. Cartoons were counterbalanced with a jittered interstimulus interval (ISI) of 2000, 4000, and 6000 ms. Task duration was 15 minutes and 4 seconds. To account for individual differences in humor detection and appreciation, subjects were instructed to respond for cartoons that they perceived as humorous or not using a button box and pressing with their right index finger. Only those that were rated as humorous (among the 30 humorous trials) and not humorous (among the 40 non-humorous trials) were included in the fMRI data analyses, but the results were similar even when all trials were included.

### Post-Scan Ratings

To assess the degree of funniness (humor intensity), subjects were asked to rate each cartoon he or she found as humorous on a scale of 0 to 10, with 0 being not humorous, 1 being least humorous and 10 as most humorous. Cataplexy patients were also instructed to report any cataplexy symptoms they experienced for each trial on a scale of 0 to 10, with 0 indicating no symptoms and 10 indicating full-paralysis or inability to speak (i.e., symptoms directly related to having a cataplectic attack).

### fMRI Acquisition and Analyses

#### Acquisition

Scanning parameters used were similar to previous studies [4], [5], [8]. The scanner uses an LX platform with gradients in “Mini-CRM” configuration (35 mT/m, SR 190 mT/m/s) and includes a Magnex 3-Tesla 80 cm magnet. Images were acquired on a 3-Tesla GE Signa scanner using a standard GE whole-head coil. 28 axial slices (4 mm thickness, 0.5 mm skip) parallel to the anterior and posterior commissures covering the whole brain were imaged with a temporal resolution of 2 s using a T2* weighted gradient echo spiral pulse sequence: repetition time (TR) = 2000 ms, echo-time (TE) = 30 ms, flip angle = 80° and 1 interleave [28]. The field-of-view (FOV) was 200×200 mm and the matrix size was 64×64, giving an in-plane spatial resolution of 3.125 mm. A head restraint system was applied to prevent head movements due to laughter. Scan and task synchronization was achieved using a TTL pulse distribution to the scanner timing microprocessor board from a button box linked to a G3 Macintosh. An automatic shim was applied to maximize homogeneity of the magnetic field.

#### Preprocessing

Inverse Fourier Transform was used to reconstruct images for each of the 450 frame time points into 64×64×28 image matrices (voxel size: 3.75×3.75×4 mm). Statistical parametric mapping 2 (SPM2, http://www.fil.ion.ucl.ac.uk) was used to preprocess all fMRI data. Data were slice time corrected, realigned to the reference volume, normalized using an echo-planar imaging template into Montreal Neurological Institute (MNI) stereotactic space and resampled to 2×2×2 mm voxels using trilinear interpolation, and finally smoothed at a full-width-half-maximum of 4 mm to reduce spatial noise.

#### Individual Statistics

Voxel-wise activations during humorous compared to non-humorous cartoon trials, defined by button box responses collected during the scanning session were analyzed for each subject using a fixed effects model. A high pass filter (120 sec/cycle) was applied to remove confounding effects of fluctuations in global mean. A regressor waveform for each condition, convolved with a 6 second delay Poisson function accounting for delay and dispersion in the hemodynamic response function, was used to compute voxel-wise t statistics, which were then normalized to z scores to provide a statistical measure of activation that is independent of sample size.

#### Within Group Statistics

Images comparing Humorous trials against Non-Humorous trials (Humorous>Non-Humorous, one per subject) were submitted to a random effects model to determine brain regions that showed greater activation during humorous compared to non-humorous events. One-sample t-tests using these Humorous>Non-Humorous comparison images were used to determine within group activation. Significant clusters of activation were determined using the joint expected probability distribution with dual height and extent threshold (p<0.01) corrected at the whole brain level.

#### Between Group Statistics

Two-sample t-test was used to determine significantly different brain activation patterns between the Control and Cataplexy groups. Specifically, we examined regions that showed significantly different activation between the Cataplexy and Control groups. Gender is known to influence brain activation in brain regions involved in humor appreciation [8] and we had a insignificant but a slightly larger proportion of females in our Cataplexy group as a result of having to exclude two subjects from the group analyses (hence reducing the subjects from 12 to 10). Therefore we repeated the between-group analyses by performing analyses of covariance entering gender as a nuisance variable and obtained similar results.

Activation maps were superimposed on high-resolution T1-weighted images and coordinates of activation were converted from MNI to Talairach space using the mni2tal function (http://www.mrc-cbu.cam.ac.uk/Imaging/Common/mnispace.shtml). Locations interpreted using universal neuroanatomical landmarks 29–31.

Time series from the hypothalamus, nucleus accumbens, and right inferior frontal gyrus were extracted for each subject and mean averages for the Humorous and Non-Humorous conditions were plotted (Fig. 1d). Regions were determined from the Humorous>Non-Humorous comparison in a one-sample t-test pooling all subjects (p<0.05 small volume corrected, using regions of interest from [32]).

#### Single Case Analysis

*Of A Cataplectic Attack.* A full cataplectic attack occurred during a cartoon rated as humorous on the 63^rd^ trial (Attack trial) in one subject (Attack patient). To investigate the neural basis underlying this cataplectic attack, a general linear model was created with an additional comparison modeling the attack trial. All trials after the attack were not modeled. Time-series analyses were performed similarly to other subjects except that we additionally examined the attack trial to other trials for this subject, to other subjects in the Cataplexy group, or to the Control group for particular regions-of interest.
